# Molecular epidemiology of a hepatitis C virus epidemic in a haemodialysis unit: outbreak investigation and infection outcome

**DOI:** 10.1186/1471-2334-10-257

**Published:** 2010-08-27

**Authors:** Simone Lanini, Isabella Abbate, Vincenzo Puro, Fabrizio Soscia, Franceso Albertoni, Walter Battisti, Amilacare Ruta, Maria R Capobianchi, Giuseppe Ippolito

**Affiliations:** 1 Istituto Nazionale per le Malattie Infettive Lazzaro Spallanzani via Portuense 292 00149 Rome, Italy; 2 Azienda Unità Sanitaria Locale Latina, Centro Direzionale Commerciale Latinafiori, Viale P.L.Nervi snc, 04100 Latina Italy

## Abstract

**Background:**

HCV is a leading cause of liver chronic diseases all over the world. In developed countries the highest prevalence of infection is reported among intravenous drug users and haemodialysis (HD) patients. The present report is to identify the pathway of HCV transmission during an outbreak of HCV infection in a privately run haemodialysis (HD) unit in Italy in 2005.

**Methods:**

Dynamics of the outbreak and infection clinical outcomes were defined through an ambi-directional cohort study. Molecular epidemiology techniques were used to define the relationships between the viral variants infecting the patients and confirm the outbreak. Risk analysis and auditing procedures were carried out to define the transmission pathway(s).

**Results:**

Of the 50 patients treated in the HD unit 5 were already anti-HCV positive and 13 became positive during the study period (AR = 28.9%). Phylogenic analysis identified that, all the molecularly characterized incident cases (10 out of 13), were infected with the same viral variant of one of the prevalent cases. The multivariate analysis and the auditing procedure disclosed a single event of multi-dose vials heparin contamination as the cause of transmission of the infection in 11 out of the 13 incident cases; 2 additional incident cases occurred possibly as a result of inappropriate risk management.

**Discussion:**

More than 30% of all HCV infections in developed countries results from poor application of standard precautions during percutaneous procedures. Comprehensive strategy which included: educational programmes, periodical auditing on standard precaution, use of single-dose vials whenever possible, prospective surveillance for blood-borne infections (including a system of prompt notification) and risk assessment/management dedicated staff are the cornerstone to contain and prevent outbreaks in HD

**Conclusions:**

The outbreak described should serve as a reminder to HD providers that patients undergoing dialysis are at risk for HCV infection and that HCV may be easily transmitted whenever standard precautions are not strictly applied.

## Background

Hepatitis C virus (HCV) is a blood borne pathogen which has been recognized to be one of the major causes of chronic liver disease worldwide [1].

In Italy the highest prevalence of HCV infection (usually higher than 10%) is reported among intravenous drug users, subjects who received blood coagulation factors before 1987, subjects who received blood transfusions or organ transplantation before 1992 and haemodialysis (HD) patients [2].

HCV is an important cause of morbidity and mortality in patients with end-stage renal disease [2,3], in fact, despite the introduction of anti-HCV routine screening for blood donors and the introduction of erythropoietin in the early '90 s [4,5], in Italy the prevalence of HCV among HD patients is much higher than in the general population [2,6]. Nevertheless, recent lines of evidence indicate that HCV can hardly be transmitted during dialysis procedures when state-of-the-art machines are used [7]; therefore it has been suggested that the increased incidence of infections may result from poor application of standard precautions, such as hand hygiene, proper use of gloves, and safe injection practices[8,9].

Between 27 July and 29 September 2005, 13 cases of newly acquired HCV infection occurred among the 50 end-stage renal disease patients who were cared for in a privately run HD unit in Lazio Region (central Italy). In response to this outbreak an epidemiological investigation was conducted by the National Institute for Infectious Disease "*Lazzaro Spallanzani*". The present paper reports the epidemiological investigation carried out to confirm the presence of the outbreak, to identify the source(s) of infection, to elucidate the transmission pathway(s) and to define the clinical outcome of infected patients. The report has been written according to "The ORION statement: guidelines for transparent reporting of outbreak reports and intervention studies of nosocomial infection" [10].

## Methods

### Study design

#### Retrospective cohort study

To assess potential risk factors associated with the infection a 33-week retrospective cohort study was conducted. Susceptible subjects were enrolled at the time of their first HD starting on Monday February 7 2005 and exited either on Saturday September 24 2005 (end of follow-up), the last know HD before Saturday September 24 2005 (lost to follow-up) or the day of the first anti-HCV positivity (incident cases). The date of HD and relevant patients' data (i.e.: age, gender, HBsAg status and all available data about HCV testing) were obtained either by the medical director of the unit or directly from patients' medical records. A 33-week time period was considered since it is the time when we believe transmission most likely occurred. It includes a 24-week time period before the onset first anti-HCV (we considered the earliest possible contagion date) and the end of the week before the onset of the last known case (end of outbreak).

#### Molecular epidemiology

To evaluate the presence of additional cases, to confirm the anti-HCV status of known incident/prevalent cases and to assess whether cases were infected with the same HCV molecular variant(s), all patients who were still undergoing dialysis in the HD unit on 26 September 2005 were sampled to be tested for anti-HCV and HCV-RNA. HCV-RNA positive samples were genotyped and, for samples with the same genotype, a molecular characterization of 2 different genomic regions (i.e. NS5B and HVR-1) was performed. Serum was sampled at the HD unit and sent at the virology unit of "*INMI Lazzaro Spallanzani" *to be analyzed by the second week of October 2005.

#### Prospective surveillance

All subjects that tested negative for both anti-HCV and HCV-RNA at the first test (see above) were re-tested at 12 and 24 week to confirm the end of transmission. On those occasions tests were performed locally and the results reported as soon as available by the medical director of the HD unit to the epidemiological team. For patients who were only occasionally dialyzed in the HD unit or who moved to another HD centre, information about anti-HCV status was obtained by contacting patients' HD centres staff.

#### Auditing procedure

Auditing procedure was conducted to reveal potential inadequacies in the practices of staff at the HD unit and to define possible transmission pathways. All medical records and HD treatment logs were analyzed. Application of standard precautions was assessed by interviewing all HCWs and by reviewing all internal protocols in use. Moreover infected patients were interviewed to evaluate the presence of other risk factors for HCV infection and to obtain additional information on the actual implementation of standard precautions. The HCWs' anti-HCV status was evaluated through the examination of the compulsory tests for viral hepatitis done according to the Italian health regulation [11,12].

#### Clinical follow-up of incident cases

Between September 2005 and October 2006 all incident cases were followed-up at the infectious disease unit of the "*Azienda Sanitaria di Latina Ospedale Santa Maria Goretti*". Patients underwent all the medical treatments according to their needs and independently from the present analysis. Relevant data to define clinical outcome (i.e. ALT level, HCV-RNA and therapy with PegInterferon alfa-2a) were obtained directly from the medical director of the unit.

### Participants and clinical setting

The HD unit was staffed with 7 doctors and 6 nurses on duty from Monday to Saturday on 12 HD machines. Patients were allotted to 4 different shifts, i.e.: Monday, Wednesday and Friday mornings (MWF-am) or afternoon (MWF-pm), Tuesday, Thursday and Saturday morning (TTS-am) or afternoon (TTS-pm).

#### Case definition

Susceptible subject: a subject anti-HCV negative at his/her last test before Monday 7 February 2005*.

Prevalent case: a subject already known to be anti-HCV positive by Monday 7 February 2005*.

Incident case: a susceptible subject who eventually became anti-HCV positive.

Possible case: an incident case without sufficient data to define the HCV molecular variant (i.e. genotype and characterization of both NS5B and HVR1 sequences)

Confirmed case: an incident case infected with the same HCV molecular variant of another incident or prevalent case (i.e. identical genotype, NS5B and HVR1 sequences).

Index case: a prevalent case infected with the same HCV molecular variant of another incident case (i.e. identical genotype, NS5B and HVR1 sequences).

Excluded case: prevalent or incident cases for whom molecular analysis (either genotype, NS5B or HVR1 sequences) excluded relation with at least another incident or prevalent case.

*According to the internal infection control protocols, all patients underwent anti-HCV test at the time of first HD and then every 3 months (see Results "Audit procedure").

#### Epidemiological and clinical outcomes definition

Standard precautions (which include hand hygiene, proper use of gloves, and safe injection practices) are defined according to Health Care Infection Control Practices Advisory Committee 2007 [8].

Attack rate was calculated as the proportion of incident cases over susceptible subjects at the end of outbreak.

Clinical attack rate was calculated as the proportion of incident cases with ALT > 80 UI/ml at least once after the first positive anti-HCV test out of the exposed susceptible subjects at the end of the outbreak.

Clinical outcome: incident cases were classified as either recovered, if HCV-RNA was undetectable, or chronically infected, if HCV-RNA was detectable, by the end of October 2006.

### Virological methods

#### Standard virology (HCV serology and PCR for HCV-RNA)

A third-generation immunoenzymatic assay (Axsym HCV version 3.0, Abbott Laboratories) was used for anti-HCV antibody testing. A commercially available quantitative polymerase chain reaction (PCR) assay was used to measure HCV-RNA (Amplicor HCV Monitor 2.0, Roche Diagnostics).

#### HCV genotype analysis and molecular characterization of NS5B and HVR-1

A line probe assay (InnoLipa HCV II, Bayer Diagnostics) was used to genotype HCV in all HCV-RNA positive subjects. Samples from patients with the same HCV genotype were eligible for molecular analysis of NS5B and HVR1. HCV-RNA was extracted from serum samples using the QIAamp Viral RNA kit (QIAGEN, Hilden, Germany), underwent retrotranscription by random hexamer extension and was used to perform molecular analysis. Two-strand direct sequencing was carried out on nested PCR products obtained from the NS5B region and from the hypervariable region 1 (HVR1) encompassing in the E2 gene, as previously reported [13]. Sequencing was performed on ABI Prism 3100, using the BigDye Terminator cycle sequencing kit (Applied Biosystems, Warrington, UK). The sequences, aligned with CLUSTAL W software (version 1.5), were confronted by BLAST with sequences from the National Center for Biotechnology Information (NCBI) database (U.S. National Library of Medicine, Bethesda, MD, http://www.ncbi.nlm.nih.gov), to confirm the genotype assignment. Phylogenetic trees were constructed using the neighbor-joining method, including NS5B and HVR-1 reference sequences from GenBank, as well as sequences of genotype 2c obtained from routine Laboratory diagnostics or from previously analyzed clusters of transmission, all epidemiologically unrelated to the described episode (see figure legend). All the algorithms used are included in the Mega package (version 2.1). The results of this analysis were used to confirm a monophyletic cluster of infection.

### Statistical methods

Overall and stratum specific rates with 95% confidence interval (95% CI) were calculated as cases per 100 dialysis-week person. Crude and adjusted rate ratios (RR), 95% CI interval and p-value of dichotomous variables were calculated according to Poisson regression with a robust error variance for binary data as described by Zou G. [14]. Discrete variables were assessed with a conventional Poisson regression. The following were considered as potential risk factors: age >60, gender, having been dialyzed at least once either after or along a HCV genotype 2c positive subject, overall number of HD while anti-HCV negative (as the only discrete variable), being usually dialysed either on MWF-am or MWF-pm or TTS-am or TTS-pm or other (each patient was allocated in one of the 5 above group according to the shift he/she had 75% or more HD or "other" if this criterion was not met for any shift). A multivariate model was built up including only the risk factors with a p-value less than 0.10 from the univariate analysis. Statistical significance was assumed if p < 0.05. The statistical analysis was performed by using STATA 11 statistical package (Stata-Corp, 4905 Lakeway Drive College Station, Texas 77845 USA).

## Results

### Retrospective cohort study

**Figure 1:**
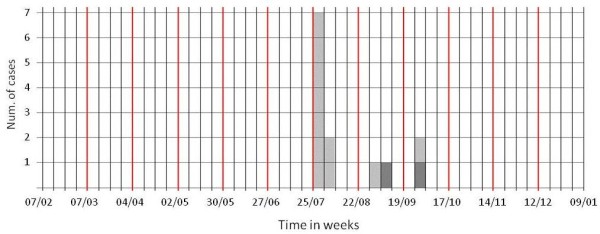
**Epidemic curve indicating the week when each of 13 incident cases was identified (i.e.: first anti-HCV positive test)**. In particular 7 cases where reported on 27 July, 2 on 3 August, 1 on 30 August, 1 on 8 September 1 on 27 September and 1 on 28 September (case occurred on 27 and 28 September were reported on the same week). Light grey squares indicate cases who underwent HD in the afternoon shift of the 20 April 2005. Dark grey squares are for subjects who did not undergo HD in the afternoon shift of the 20 April 2005. Red lines define a four week periods.

Between 7 February and 24 September 2005, fifty patients underwent HD in the unit. Data on HBsAg and anti-HCV were available for all of them. The main epidemiological characteristics of the patients are shown in Table 1. Out of the total 50 patients, 45 were susceptible subjects and 5 were prevalent cases. By the end of September 2005, 13 of the 45 susceptible subjects became anti-HCV positive (incident cases), with a crude overall incidence rate of 1.07 cases per 100 dialysis-week person. Incident cases that occurred by 30 August (10 cases) had also available HCV genotype determination which showed that all of them were infected with genotype 2c HCV (figure 1).

**Table 1:** Epidemiological feature of the 50 subjects who underwent dialysis during the 33 weeks considered in the retrospective analysis.

Characteristics	Number
Total	50

Female (%)	14 (28%)

Age (IQR)	63 (47-71)

Median number of dialysis per patient (range)	98 (1-125)

Median dialysis per week (IQR)	2.97 (2.87-3.00)

Anti-HCV positive (%)	5 (9.8%)

HBsAg positive (%)	1 (1.9%)

IQR = inter-quartile rage

Univariate analysis of retrospective patients' data gave strong evidence of association between being an incident case and being usually dialyzed on MWF-pm shift (p < 0.001; RR 7.79 [2.91 - 20.86]). A lesser degree of evidence was found between being an incident case and having been dialysed at least once either along (p = 0.033; RR = 0.27 [0.08-0.89]) or after (p = 0.090; RR = 0.27 [0.82-14.53]) a HCV genotype 2c positive subject. The multivariate model confirmed that only undergoing HD on MWF-pm shift was independently associated with being an incident case (p = 0.019; RR 10.00 [1.47-68.02]); in fact, 9 out of the 13 incident cases used to have HD in this shift (table 2).

**Table 2:** Results of the retrospective cohort study for the analysis of risk.

Risk factor		Cohort description				Univariate analysis		Multivariate analysis	
		
		Subjects	Cases	**Time at risk**^**A**^	**Rate**^**B **^**(95%-CI)**	RR (95%-CI)	**p-value**^**C**^	RR (95%-CI)	**p-value**^**C**^
Age > 60	No	16	7	428.86	1.63(0.78-3.42)	0.47	0.121	-	-
	Yes	29	6	781.71	0.77(0.34-1.71)	(0.18-1.22)			

female sex	No	32	9	869.57	1.03(0.54-1.99)	1.13	0.835	-	-
	Yes	13	4	341.00	1.17(0.44-3.13)	(0.40-3.22)			

MWF-am	No	36	12	947.00	1.27(0.72-2.23)	0.30	0.218	-	-
	Yes	9	1	263.57	0.38(0.05-2.69)	(0.04-2.04)			

MWF-pm	No	34	4	939.14	0.43(0.16-1.13)	7.79	**< 0.001**	**10.00**	**0.019**
	Yes	11	9	271.43	3.32(1.73-6.37)	(2.91-20.86)		**(1.47-68.02)**	

TTS-am	No	36	12	956.14	1.26(0.71-2.21)	0.31	0.236	-	-
	Yes	9	1	254.43	0.39(0.06-2.79)	(0.05-2.14)			

TTS-pm	No	34	12	936.29	1.28(0.73-2.26)	0.28	0.202	-	-
	Yes	11	1	274.28	0.36(0.05-2.59)	(0.04-1.96)			

Other	No	40	12	1063.71	1.13(0.64-1.99)	0.60	0.626	-	-
	Yes	5	1	146.86	0.68(0.1-4.83)	(0.08-4.60)			

Dialysis after gen2 HCV+	No	23	2	467.57	0.43(0.11-1.71)	3.46	0.090	1.29	0.658
	Yes	22	11	743.00	1.48(0.82-2.67)	(0.82-14.53)		(0.13-12.65)	

Dialysis along gen2 HCV+	No	18	10	578.29	1.73(0.93-3.21)	**0.27**	**0.033**	1.70	0.804
	Yes	27	3	632.28	0.47(0.15-1.47)	**(0.08-0.89)**		(0.16-18.10)	

N. of dialysis	-	-	-	-	-	0.99 **^D^**	0.291	-	-
						(0.97-1.01)			

Overa all	-	45	13	1210.57	1.07	-	-	-	-

Forty-five susceptible subjects underwent HD throughout the 33 weeks considered, among them 13 became incident cases with and overall crude rate of 1.07 events per 100 dialysis-week/person. First column risk factor analysed, second column descriptive analysis, third column univariate modified Poisson regression analysis of risk, fourth column multivariate modified Poisson regression. Statistically significant value (p < 0.05) in bold.

**A) **Time at risk is calculated as week of dialysis person of susceptible subjects; **B) **the rate is calculated as the number of events per 100 dialysis-week/person; **C) **p-value is calculated according to Robust standard error; **D) **estimate increment of risk for each additional HD shift (measure of association are according conventional Poisson regression). **RR **= rate ratio; **CI 95% = **95% confidence interval; **gen2 HCV + **= genotype 2 hepatitis C virus infected subject; **MWF-am **= Mondays, Wednesdays and Fridays morning dialysis shift **MWF-pm **= Mondays, Wednesdays and Fridays afternoon dialysis shift **TTS-am **= Tuesdays, Thursdays and Saturdays morning dialysis shift **TTS-pm **= Tuesdays, Thursdays and Saturdays afternoon dialysis shift.

On this basis we performed a specific analysis on MWF-pm shift only. This analysis showed that all the 11 susceptible subjects who underwent HD on 20 April eventually developed HCV infection. Analysis of data from both shifts performed on 20 April showed that overall 20 patients underwent HD in this day (9 in the morning and 11 in the afternoon). Among them was a 81 year old female patient, already known to be a prevalent case infected with genotype 2c HCV, who was dialysed in the morning. As reported in her clinical sheets, she suffered from thrombosis at her arteriovenous fistula, lost blood and received several additional doses of heparin. In the subsequent shift, all the 11 patients who underwent HD (9 as habitual and 2 as occasional patients) developed a HCV infection.

### Molecular epidemiology

Of the 50 patients dialysed between Monday February 7 and Saturday September 24 2005, forty-three (13 incident cases, 2 prevalent cases and 28 susceptible subjects) were still undergoing dialysis in the HD unit by the end of September 2005 and were sampled. We could not obtain serum samples for the 7 remaining patients (3 prevalent cases and 4 susceptible subjects). None of the 28 susceptible subjects resulted anti-HCV and/or HCV-RNA positive. All 13 incident cases and the 2 prevalent cases sampled were anti-HCV. Genotype analysis was possible for 10 incident cases and for both prevalent cases. Three incident cases (possible cases) had inadequate viral load (< 615 IU/ml) for genotype analysis. The genotyping confirmed that 1 prevalent case and 10 incident cases were infected by a same genotype 2c HCV while the other prevalent case was infected with an unrelated genotype 1b HCV and was not further investigated (excluded case). The complete characterization of the HVR-1 and NS5B regions of viral genome was performed for 8 out of 13 incident cases and for the prevalent case infected with genotype 2c. Due to low viral load (HCV-RNA 650 UI/ml and 10,996 UI/ml) 2 incident cases were characterized for NS5B genomic region only.

**Figure 2:**
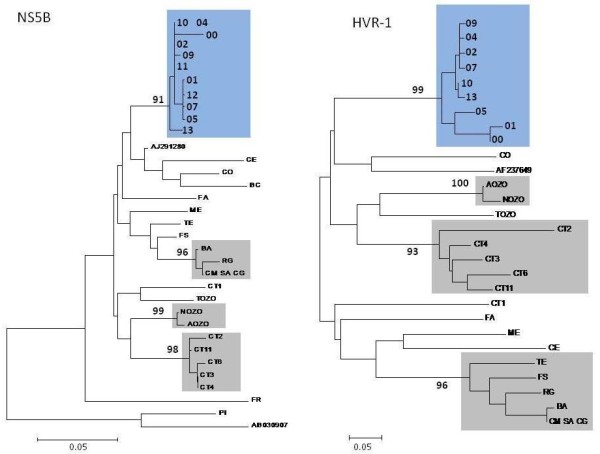
**Phylogenetic tree analysis of NS5B and HVR1 region of HCV**. The analysis of NS5B was performed for 10 incident cases and the index case (all genotype 2c). The analysis of HVR1 was possible for 8 incident cases only. Molecular analysis was not performed for 3 incident cases due to low viral load (< 615 UI/ml). Blue boxes include the viral variants from subjects in the current outbreak; each code refers to only one subject. Code 00 indicates the index case. Code 02, 04, 07, 09, 10, and 13 are confirmed cases dialysed on 20 April afternoon shift. Code 01 and 05 are confirmed cased who did not undergo dialysis on 20 April. Code 11 and 12 are possible cases (analysis of HVR-1 not available) dialysed on 20 April afternoon shift. Gray boxes include viral variant from other unrelated outbreaks.

As shown in figure 2, results of molecular analysis confirmed that the HCV genotype 2c prevalent case (index case) and the 8 incident cases (confirmed cases) characterized both for N5SB and HVR-1 represented a significant monophyletic cluster of infection. In addition the 2 incident cases (possible cases) characterized for only one genomic region shared an identical N5SB sequence with the main cluster.

### Prospective surveillance

None of the 28 susceptible subjects who were still undergoing dialysis in the unit by the end of September 2005 resulted anti-HCV or HCV-RNA after 12 and 24 week of follow-up.

We contacted attending doctors of the 4 susceptible subjects who underwent HD in other centres who confirmed that none of them showed evidence of anti-HCV and/or HCV-RNA in the same time periods. We could not obtain any data about the genotype of the 3 prevalent cases who were not sampled for the molecular epidemiology analysis.

### Audit procedure

According to the internal infection control protocols, all patients were scheduled to undergo routine serological test for viral hepatitis (i.e.: anti-HCV, anti-HBs, anti-HBc and HBsAg) and transaminases, at the time of first HD and then every 3 months. All HCWs should use personal protective equipment (PPE) such as gloves, goggles and gown depending on the procedure to be done and they should use new PPEs for each subsequent patient. The unit was not provided with protocols for risks management. HD machines were provided with disposable dialyzer circuits and electronic fail-safe systems. Apart from HBsAg positive patients, all patients (including HCV positive ones) were dialyzed in the same room with common machines.

Doctors' duties consisted in clinical evaluation of the patients before starting HD, connecting/disconnecting patients and assisting them in case of emergency. Nurses' duties consisted in taking care of the room, setting up machines, preparing drugs and looking after patients' needs throughout the HD shift. Neither the doctors nor the nurses were assigned to specific tasks or patients while on duty; the presence of 2 doctors and 2 nurses was guaranteed on each HD shift. Shifts lasted 6 hours but double-shifts (i.e. 12 h on duty) were frequent both for nurses and for doctors.

Patients used to undergo dialysis on the same shift; however shift exchanges between habitual patients and HD of occasional patients occurred throughout the period.

The interviews with HCWs (7 doctors and 6 nurses) confirmed that drugs in multi-dose vials (i.e. 20 ml heparin vials and 250 ml saline solution vials) were used in the unit. In particular, it was reported that the heparin doses to be used in one shift (i.e.: 20 ml syringe containing 18 ml saline solution and 2 ml heparin) were prepared by nurses while caring for patients on the prior shift. Preparation took place in the same room where the patients were dialyzed with a shared saline solutions and heparin vial. Neither the saline solution nor the heparin vials were disposed until exhausted; the same vials were used both to prepare drugs for the next shift and to care for patients undergoing HD. Each saline solution and each heparin vials were enough to prepare a maximum of 13 and 10 doses respectively.

Interviews with patients (9 out 13 incident cases) revealed low levels of standard precautions. In particular HCWs made numerous passages from one patient to another or to a HD machine or a keyboard without changing gloves or without hand-washing, especially during busy periods or emergencies.

By the third week of August 2005 incident cases were spread in various shifts, as a consequence of the decision of the head of the unit to reserve dedicated HD machines to HCV patients.

The HCWs' anti-HCV status was evaluated through the examination of the compulsory tests for viral hepatitis. We analyzed the results of all these tests and found that all HCWs were regularly tested every year and that none of them had ever resulted anti-HCV positive the year before and the year after the event.

### Clinical follow-up

ALT determinations during acute infection were available for all the 13 incident cases. Among them 10 (i.e.: 76.9% [46.2%-95.0%]) showed ALT >80 IU at least once while 3 never showed elevated ALT. A complete one year follow-up was possible for 12 out 13 incident cases, as one subject moved to another city by the end of October 2005. Of the 12 followed cases, 4 were already HCV-RNA negative by the end of June 2006 while 8 developed chronic infection (i.e.: chronic rate 66.7% [34.9%-90.1%]). Three of these patients underwent a 24-week mono-therapy course with PegInterferon alfa-2a (Pegasys) showing undetectable HCV-RNA levels at the end of treatment. At the end of follow-up, 7 out 12 patients followed up recovered while 5 developed a chronic infection with normal ALT levels.

## Discussion

The outbreak was suspected on the ground of a peculiarly high incidence of anti-HCV sero-conversion between July and the September 2005 and eventually confirmed through the retrospective cohort study and molecular characterization of HCV molecular variant.

This investigation showed that the 13 incident cases occurred by the end of September 2005 and for 10 of them HCV genotype was available in the clinical records (all genotype 2c). The Poisson regression which included all the 45 susceptible subjects enrolled in the retrospective study found that undergoing HD on MWF-pm shift was the only independent factor associated with the infection. The subsequent analysis of patients' flow in this shift showed that all the 11 susceptible subjects who underwent HD on 20 April afternoon eventually became incident cases. Having analyzed the medical activities reported on the logbook for that day, we found that a prevalent case (index cases) underwent HD in the morning; she had several problems with her arterovenous fistula, bled and needed several additional doses of heparin. According to this evidence and the data obtained from the audit, we hypothesised that the 11 susceptible subjects who underwent HD in the subsequent shift were infected through contaminated heparin doses prepared during the morning by the HCWs who were simultaneously involved in caring after the index case and preparing the heparin doses for the next shift. It is possible that, during the care of the index case, shared 250 ml saline solution(s) and/or shared 20 ml heparin vial(s) were contaminated by repeated drawings and injections of materials and then re-used to prepare the heparin doses for the afternoon shift. It is also possible that during the bleeding emergency the area where the heparin doses were prepared was contaminated by HCWs who failed to change gloves after caring for the index case.

Of these 11 incident cases 6 were confirmed by the molecular analysis of both NS5B and HVR-1 viral region, 2 were proved to be infected with a genotype 2c HCV with an identical NS5B sequence to the index case and other confirmed cases while for 3 incident cases we know they were infected with genotype 2c HCV by data recovered from their clinical records.

Two additional incident cases did not attended the 20 April HD. We cannot clearly define how these patients contracted the infection but there is a possibility they might have been infected as a result of: lacks of standard precautions, the increased prevalence of infection occurred after the onset of the first cluster of infections and the decision of spreading the incident cases over different shifts instead of cohorting them. The analysis of NS5B and HVR-1 viral region confirmed that both these case were infected with the same HCV molecular variant of the index cases and all other confirmed cases.

The end of transmission was defined according the results of the prospective surveillance which showed no more incident cases at 12 and 24 weeks after the onset of the last incident cases. The possibility of HCWs-to-patient transmission was ruled out since no HCW was found to be anti-HCV positive. Table 3 reports the estimated overall outcome data according to this hypothesis.

**Table 3:** This table shows the summary outcome at the end of the outbreak

	Overall outcomes
Date of detection of first and last case	27 July 2005 - 29 September 2005

Estimated date of first infection	20 April 2005

Number of susceptible subjects	45 (50 patients on HD 5 of whom already anti-HCV positive)

Number of clusters	1 main cluster plus 1 or 2 secondary events

Overall number of cases	13 (8 confirmed 5 possible)

Attack rate	28.9% (13/45)

Clinical attack rate	22.2% (10/45)

Fatalities	None

Index case	Female 81 with chronic HCV infection and end stage renal disease

Clinical outcome	5 chronic infections (none of whom was treated); 8 recovered (3 received Peg-IFN-2a therapy).

Associated factors	Lack in application of standard precautions (i.e. use of shared heparin and saline solution vials and possibly fail in changing gloves) and lack in risks management.

Several mechanisms such as: reuse of dialysis filters, internal contamination of HD machines and contamination of environment, can potentially contribute to patient-to-patient HCV transmission in HD unit. However lack of standard precautions, including hand-washing, proper use of gloves and safe injection practices, have been recognized as leading causes of HCV outbreak in HD settings [7,8,15-19].

HCV transmission is a major concern in HD units in Italy [2] and other developed countries [8,15,16,18,19]. In fact, the close proximity of patients who require frequent vascular access and high prevalence of chronic HCV carriers in this population may enhance the chance of cross-infections when standard precautions are not strictly applied. Savey et al described a large outbreak of 61 patients in a French HD centre mainly supported by patient-to-patient transmission via HCWs' hands [18]. In addition a recent systematic review on transmission of HCV in healthcare settings in USA between 1998 and 2008 reported that inappropriate use on multiple patients of single use vials and failure to store and prepare medications under aseptic conditions were responsible for HCV transmission in 15 outbreaks occurring in nonhospital healthcare settings (6 of which were HD units)[16]. As a consequence, to prevent transmission of blood borne viruses in HD settings, Centers for Diseases Control and Prevention (CDC) recommend that all single-use injectable medications and solutions should be dedicated for use on a single patient and should be entered one at a time only [20].

In a recent paper CDC's investigators outline one possible way in which inappropriate use of medication vials intended for single-person may result in large HCV outbreaks. Through direct observation they found that HCWs used to draw medication from a single-use vial of propofol with a sterile needle. The medication was injected directly through an intravenous catheter into the patient's arm. If a patient required more sedation, the needle was removed from the syringe and replaced with a new needle; the new needle with the old syringe was used to draw more medication. Backflow from the patient's intravenous catheter or from needle removal contaminated the syringe with HCV and subsequently contaminated the vial [21]. A similar transmission pathway has been reported by Germain et al [17] and could have occurred in the present outbreak. An additional factor that may have contributed to the spreading of the infection was that medication were not handled, as recommended [22], in a clean separated area, in fact, heparin doses were prepared by HCWs, while attending a HD shift and in the same room where the patients were dialyzed.

Another important flaw we found in the organization was that the unit was not provided with any protocol for risk management nor was a single HCW appointed to act as risk manager so that, the alert to local health authority was given only by September 2005, while the first cases had already occurred by the end of July 2005. In addition at the onset of the outbreak, incident cases were allocated in different shifts, a decision likely to have favoured the occurrence of the 2 additional cases. With regard to the importance of this latter issue, a topical Canadian experience in dealing with the recent lethal *Clostridium difficile *outbreaks emphasizes that a comprehensive system of risk management including dedicated staff and prompt notification of cases was the single most important factor in containment of ongoing epidemics and in preventing new ones[23].

Although data is limited, this investigation suggests that, among end-stage renal disease subjects, HCV acute infection is not much more severe than acute infection in the general population, as no patient developed fulminant hepatitis and the 38.4% (5 out of 13) spontaneous recovery rate we found was not lower than the rate of spontaneous viral clearance reported in the general population[1].

The limitations of this study are that a specific mode of transmission could not be identified for 2 of the 13 patients. Moreover since HCV-RNA testing was performed only after the onset of the outbreak and the transaminases were tested only every 3 months, we could not define the exact time between the infection and the onset of viremia and/or the liver injury which would have been precious to further describing the natural history of HCV infection in subjects with end stage renal disease. In addition we could not perform a comprehensive investigation of the unit, so that the potential role of the contamination of HD machines and of the environment was not completely evaluated. Finally potential misclassification of the 5 possible cases may have occurred since it cannot be completely ruled out that these subjects were part of other cluster(s) trigged by one or more of the three prevalent cases without molecular characterization. Nevertheless we consider this latter hypothesis quite unlikely since all possible cases underwent HD on 20 April afternoon and 2 of them shared an identical genotype and N5SB with the other confirmed cases.

## Conclusions

Apart from illicit drug addiction, medical procedures are the most important cause of HCV infection in developed countries. It has been estimated that in western countries more than 30% of all HCV infections results from poor application of standard precaution during percutaneous procedures [24]. The outbreak described here should serve as a reminder to HD providers that patients undergoing dialysis are at risk for HCV infection and that HCV may be easily transmitted whenever standard precaution, including safe injection practices and proper use of gloves are not strictly applied [25]. We believe that a comprehensive strategy including: educational programmes, periodical auditing on standard precaution, use of single-dose vials whenever possible, prospective surveillance for blood-borne infections (including a system of prompt notification) and risk assessment/management dedicated staff are the cornerstone to contain and prevent outbreaks in HD.

## Abbreviations

**ALT**: alanine transaminases; **Anti-HBc**: anti hepatitis B core antigen antibody; **Anti-HBs**: anti hepatitis B surface antigen antibody; **Anti-HCV**: anti hepatitis C antibody; **95% CI**: 95% confidence interval; **HBsAg**: hepatitis B surface antige; **HCV**: hepatitis C virus; **HCV-RNA**: anti hepatitis C ribonucleic acids; **HCW**: healthcare worker; **HD**: haemodialysis; **HVR1**: hepatitis C hyper variable region 1; ml: millilitre; **MWF-am**: Mondays, Wednesdays and Fridays morning haemodialysis shift; **MWF-pm**: Mondays, Wednesdays and Fridays afternoon haemodialysis shift; **NS5B**: hepatitis C non structural protein 5B; **PPE**: personal protective equipment; **RR**: rate ratio; **TTS-am**: Tuesdays, Thursdays and Saturdays morning haemodialysis shift; **TTS-pm**: Tuesdays, Thursdays and Saturdays afternoon haemodialysis shift; **UI**: international unit.

## Competing interests

The authors declare that they have no competing interests.

## Authors' contributions

SL made substantial contributions to the conception and design of the study, acquisition, analysis and interpretation of data, was involved in drafting the manuscript and gave approval of the final version. IA and MRC made substantial contributions to the analysis of biological specimens for virological and molecular epidemiology were involved in critically revising the manuscript for important intellectual content and gave approval of the final version. VP, FS, FA, AR, WB, and GI made substantial contributions to the analysis and interpretation of data, critically revised the manuscript for important intellectual content and gave approval of the final version.

## Pre-publication history

The pre-publication history for this paper can be accessed here:

http://www.biomedcentral.com/1471-2334/10/257/prepub
